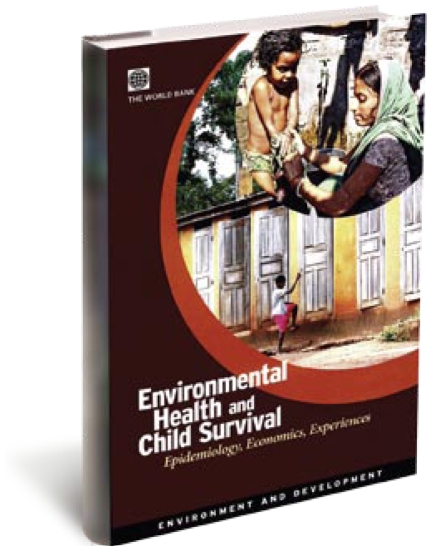# Environmental Health and Child Survival: Epidemiology, Economics, Experiences

**Published:** 2009-10

**Authors:** Leonardo Trasande

**Affiliations:** Leonardo Trasande is assistant professor of community and preventive medicine and of pediatrics at Mount Sinai School of Medicine

As the developing world industrializes, it is likely to experience an epidemiologic transition not unlike what has occurred in the United States and other countries in the developed world, with increasing prevalence and incidence of chronic conditions such as asthma and neurodevelopmental disorders. Environmental prevention has been proven to reduce childhood morbidity, yet interventions to abate environmental hazards can be quite costly. Economic data on the costs of these conditions—and the failure to prevent them, which is ultimately borne by society—may persuade policy makers that the benefits of proactive reductions in toxic environmental exposures are worth the investment.

It is within this context that the Environmental Health Anchor Program of the World Bank has developed analyses that document the burden of environmentally mediated diseases in developing countries. These analyses build on groundbreaking work by the World Health Organization (WHO) to estimate the global- and country-specific burden of disease that can be attributed to environmental factors. The authors quantify the economic costs that can be attributed to a subset of the environmental factors that the WHO has analyzed—urban air pollution, indoor air pollution, and water sanitation/hygiene. They also quantify direct and malnutrition-mediated health consequences of poor water, inadequate sanitation, improper personal/household hygiene, and inadequate water resource management.

The World Bank team compiled data for Ghana and Pakistan as two illustrative examples, quantifying mortality from pneumonia/acute lower respiratory illness, diarrhea, and malaria as well as prevalence data for diarrhea and malnutrition. They then applied WHO environmentally attributable fractions for these conditions, and applied a human capital approach to quantify lost wages that result from environmental risk factors. Although past studies have assessed the direct impact of these factors, this project also examines indirect consequences of increased malnutrition due to environmental factors—an underrecognized yet significant burden on society that had not yet been incorporated. The direct and indirect costs associated with environmental risk factors in Ghana and Pakistan are 9.3% and 8.8%, respectively, of these countries’ gross domestic product (GDP). The authors estimated that 35,702 deaths in Ghana and another 187,429 in Pakistan annually can be attributed to environmental risk factors for the health problems studied. Adding educationally derived lost income (> US$400 million in Ghana and > US$5.2 billion in Pakistan annually), the annual costs of these environmental risk factors total US$1.0 billion in Ghana and US$9.9 billion in Pakistan. These costs are representative of their WHO regions, and were chosen because a similar analysis was perfomed (without considering malnutrition effects) previously. The book closes with a discussion of successful initiatives in Mexico, Vietnam, and other countries against these risk factors, providing a partial roadmap for policy efforts in these regions.

The methodology of quantifying and costing educational attainment, mortality, and lost economic productivity is highly rigorous, and the text itself serves as a very good introduction to the novice who is considering a similar analysis in a different context. Detailed appendices describe for environmental health researchers or economists the data sources and methodologic justifications for the chosen inputs. The references strengthen the text and make it a worthy reference for the experienced researcher as well. The text suffers from the evolving nature of knowledge regarding specific environmental hazards, which is not a fault of the authors; but the analysis is limited to a small number of relatively well-established hazards. The economic data produced through this analysis are extremely conservative and should be considered only a starting point for more comprehensive and varied follow-up studies. As the epidemiologic transition continues and chemical exposures mount in importance, other diseases will likely arise as significant costs to society.

Policy makers should thus examine the history of the developed world in anticipating future economic costs if proactive measures are to be taken to prevent hazards not yet obvious, but perhaps already causes of subclinical toxicity to children. The estimates of percent GDP lost due to preventable factors in the environment are therefore even more conservative, yet the policy advice proposed—to place environmental health high on the development agenda and increase recognition that environmental health does not inhibit economic development—is even more poignant. However, the World Bank, WHO, United Nations, and other international entities should not focus so narrowly on the proposed interventions in its agenda. This analysis represents the lowest of low-hanging fruit of economically favorable interventions to protect the environmental health of populations in the developing world. These entities should expand their view on a broader scope of exposures, and are likely to find opportunities for great economic development through a focus on safe expansion of chemical use and management of hazards that emerge on the path to the group of developed nations.

## Figures and Tables

**Figure:**